# Torsional Tribological Behavior and Torsional Friction Model of Polytetrafluoroethylene against 1045 Steel

**DOI:** 10.1371/journal.pone.0147598

**Published:** 2016-01-22

**Authors:** Shibo Wang, Chengchao Niu

**Affiliations:** 1 School of Mechanical and Electronic Engineering, China University of Mining and Technology, Xuzhou, Jiangsu Province, 221116, China; 2 State Key Laboratory of Tribology, Tsinghua University, Beijing 100084, China; Massachusetts Institute Of Technology, UNITED STATES

## Abstract

In this work, the plane-on-plane torsional fretting tribological behavior of polytetrafluoroethylene (PTFE) was studied. A model of a rigid, flat-ended punch acting on an elastic half-space was built according to the experimental conditions. The results indicate that the shape of *T–θ* curves was influenced by both the torsional angle and the normal load. The torsion friction torque and wear rate of PTFE exponentially decreased when the torsion angle rose. The torsional torque increased from 0.025 N·m under a normal load of 43 N to 0.082 N·m under a normal load of 123 N. With sequentially increasing normal load, the value of torque was maintained. With rising normal load, the wear mass loss of PTFE disks was increased and the wear rate was decreased. Good agreement was found with the calculated torque according to the model and the experimental torque except for that under a normal load of 163 N. The difference under a normal load of 163 N was caused by the coefficient of friction. Usually the coefficient of friction of a polymer decreases with increasing normal load, whereas a constant coefficient of friction was applied in the model.

## Introduction

Torsional friction is defined as the reciprocating rotational motion of a pair under a normal load. It widely occurs in human hip joints and knee joints, sliding slewing bearings used in engineering machines, the center plate of bogies, and other reciprocating rotation parts of conveyances. Ball-on-disk torsional wear behavior has been widely researched with experimental methods involving torsional angle, normal load, environment, and friction pair material. The effect of testing parameters including angular displacement amplitudes, normal loads, and environments on the torsional fretting tribological behavior of LZ50 steel against AISI52100 steel has been investigated [1–3]. The torsional dynamic behavior and damage process was found to depend strongly on the normal loads, angular displacement amplitudes, and cycles. The mixed and gross slip regimes shifted in the direction of larger angular displacement amplitudes in nitrogen, compared to that in air, and torsional wear damage in nitrogen was more severe than in ambient air. Yu [4–5] found that the torsional torque of UHMWPE/TC4 was higher than that of UHMWPE/Al_2_O_3_ under a 100 N normal load, whereas the inverse was true under a normal load of 200 N. The friction-dissipated energy of the UHMWPE/Al_2_O_3_ contact was higher than that of the UHMWPE/TC4 contact. The study performed by Cai [6] indicated that, compared to the PMMA/PMMA contact, the boundary of the fretting regime of the PMMA/GCr15 contact shifted toward the direction of small angular displacement. More severe damage occurred in the PMMA/PMMA contact than in the PMMA/GCr15 contact under the condition of torsional fretting wear. Wear mechanisms for the PMMA/GCr15 contact include fatigue wear, abrasive wear, and oxidative wear with transfer layer formation. For the PMMA/PMMA contact, wear mechanisms were mainly abrasive wear and fatigue wear. Briscoe et al. had investigated the wear behaviors of PMMA under various contact conditions, from the torsional fretting mode to the rotational fretting mode [7–9]. These studies revealed that torsional contact was more detrimental to the wear resistance of PMMA than rotation contact and that the interfacial energy induced a preferential debris emission under the condition of torsional fretting. In the torsional contact configuration, the contact zone kinematics had a pronounced influence on the accumulation, compaction, and displacement of debris particles from the contact.

Plane-on–plane torsional fretting tribology has seldom been studied, although this contact regime often occurs in practice, such as in a sliding slewing bearing or a center plate of a bogie. The authors of this paper studied the plane-on-plane torsional wear behavior of MC nylon composites under different torsional angles [10–11]. The shape of the torque–angular displacement (*T*–*θ*) curves changed from elliptic to quasiparallelogrammatical with angular displacement increasing from 5° to 30°. Serious wear characterized by a deep groove of about 1.5–4 mm in radius occurred in the contact zone on the steel surface. The torsional contact area was divided into three zones: a central stick zone, an intermediate mixed-slipping annulus, and a peripheral sliding annulus. The most serious wear occurred in the intermediate annulus because of the higher contact stress and mixed-slipping regime. The friction coefficient of MC nylon composites was the main factor affecting the torsion contact regime, and the torsional wear behavior was influenced by the torsion contact regime.

Contact regime plays an important role in fretting tribology. Usually, the shape of friction force versus displacement (*F*-*D*) in tangential fretting and friction torque versus angular displacement (*T*–*θ*) in torsional fretting was used for analyzing the contact zone kinematics after fretting testing [1–3, 6, 12]. Jeong and Yong [13] found that the phase difference between the friction force signal and the relative displacement signal could indicate the degree of stick-slip during a ball-on-disc tangential fretting. Further, the phase difference was normalized by its maximum value to define as a signal parameter and then named as the fretting signal index. The fretting signal index can be used as a quantitative parameter for determination of the fretting contact regime [14].

To deeply understand the torsional behavior of a ball-on-disk, the contact regime have been analyzed by various researchers. Lubkin [15], Hetenyi [16], and Hills [17] analyzed the situation when the normal and torsional loads were coupled. Johnson [18] presented the solution of a sphere in torsion when a partial slip regime is established on the contact area. Gallego et al. [19–20] advanced a numerical model for the elastic contact under various loading types, and they applied it to tangential fretting, normal fretting, and torsional fretting. Grasinaru et al. [21] advanced a numerical algorithm based on the conjugate gradient method to simulate the slip-stick elastic torsional contact.

In the case of plane-on-plane torsional friction, the contact model was depicted as a flat-ended punch on a plane under both normal load and torsional load. Johnson *(18)* analyzed the stresses produced in an elastic half-space by the action of a rigid, flat-ended punch pressed into the surface and the stresses produced by a torsional torque actiing on a circular region, respectively. Jäger [22] built a consistent theory for normal and tangential contact under monotonically increasing normal load and oblique loading in the form of a superposition of incremental flat punch solutions. Qiu [23] analyzed the interface contact regime when an elastic half-space were pressed with an elastically similar flat-ended cylinder.

Although both ball-on-disk and plane-on-plane torsional behavior have been researched through experimental and analytical methods, the relation between experimental and numerical results is not clear. In this paper, the torsional tribological behavior of polytetrafluoroethylene (PTFE) under different torsional angular displacements and normal loads was studied with a plane-on-plane tester. A torsional friction model described by a rigid flat-ended punch on an elastic half-space was also built. The experimental and numerical results were analyzed and compared.

## Materials and Experiment

A commercially available PTFE polymer, in the form of a cylinder with a diameter of 50 mm and a thickness of 30 mm, was used as the flat specimens in this study. The flat specimens were polished to a roughness of 0.01 μm. The pin specimen with a diameter of 10 mm was made from 45# steel (Chinese trade name; the same as AISI1045 steel).

The torsional friction tests were performed with a self-made plane-on-plane torsional tester [10], the details of which have been described in a previous research paper. In this study, torsional wear tests were performed at a frequency of 1 Hz. The torsional angular displacement amplitudes were set as 0.5°, 1°, 2,5°, 10°, 15°, and 30°. Normal loads were selected as 43, 83, 123, and 163N, which induced normal pressures of 0.5, 1, 1.5, and 2 MPa, respectively. The test duration of torsion wear tests was 300,000 cycles. The variations of the friction torques (*T*) versus torsional angular displacement amplitude (*θ*) can be recorded as a function of the number of cycles during the torsional fretting tests. The amount of wear of the PTFE composite cylinders was measured by the wear mass loss. The PTFE samples were dried in an oven at 80°C for 8 h before and after wear testing. Then the samples were weighed by an electronic balance with an accuracy of 0.01 mg.

Flat indentation creep tests were conducted in the testing machine UMT-III at room temperature (24°C), according to the actual testing forces. It was used to study the visco-elastic properties of PTFE for a compatible contact model. The testing apparatus had a displacement resolution of 0.1μm and a force resolution of 1mN, which allowed the accurate measurements. The indentation cycle consisted of a touching part, a loading part and a hold period. The touching force was set as 1% of the maximum load. The loading force was applied linearly with the loading rate 0.5N/s until it reached the target value. Then it was followed by a stable hold period of 5h at maximum load. Experiments ware repeated twice and offered very good repeatability. Usually, during flat indentation creep testing, the material is stiffer and the subsequent relaxation is higher at high loading rates. In order to minimise the influence of loading rate, the indentation loading rate of 0.5N/s is same to the rate of normal load applied in torsion friction testing.

## Results

### Effect of torsional angle

**Fig 1:**
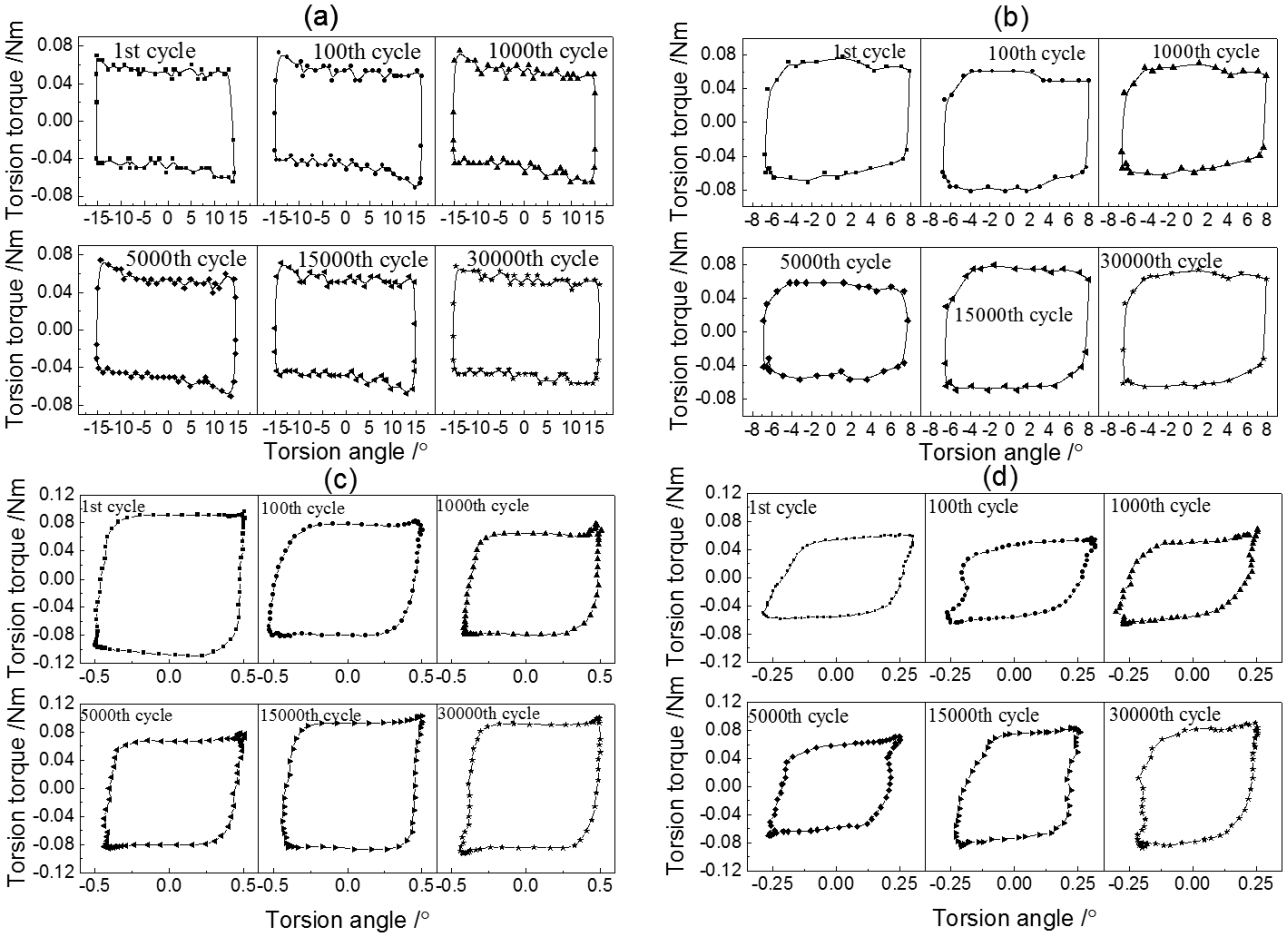
*T*–*θ* curves of PTFE under different torsion angles with a normal load of 123 N. (a) *F* = 123 N,*θ* = 30°; (b) *F* = 123 N, *θ* = 15°; (c) *F* = 123 N,*θ* = 1°; (d) *F* = 123 N, *θ* = 0.5°.

*T–θ* curves of PTFE disk against a steel pin under different torsional angle displacements with a 123 N normal load during torsional friction are presented in Fig 1A–1D. The shape of the *T–θ* curves was influenced by the torsional angle: rectangular under the larger torsional angle displacement (30° and 15°) and quasiparallelogrammatical under the smaller torsional angle displacements (1° and 0.5°), respectively. Based on the typical linear fretting map [24, 25], a slight drop in torque of pure PTFE under 30° and 15° torsional angle displacement indicated that gross slip was the main torsion kinematics behavior in the contact zone. The torsion kinematics behavior with a hysteresis quasiparallelogrammatical loop of PTFE under 1° and 0.5° was partial slip.

**Fig 2:**
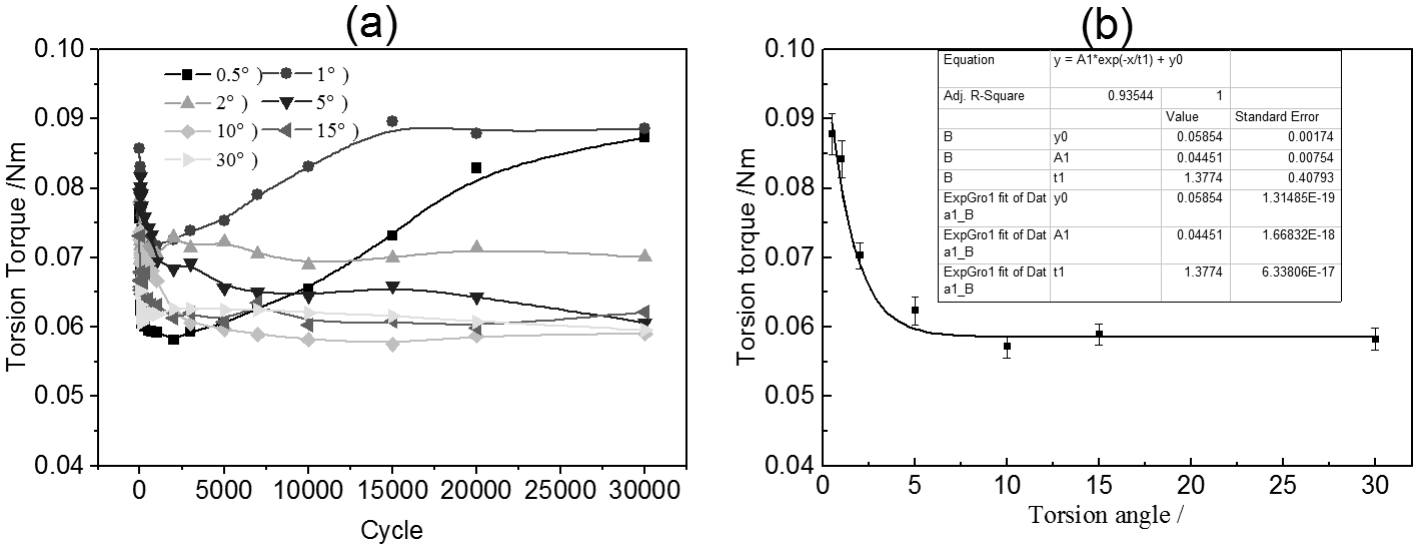
Variation of torsional friction torque of PTFE during torsional wear testing (a) Torsional friction torque versus twist cycles; (b) Torsional friction torque of PTFE versus torsion angle.

The variation of torsional friction torque of PTFE during torsional wear testing is exhibited in Fig 2A. For the smaller torsional angles (*θ* = 0.5° and 1°), the torsional friction torque first decreased quickly, then increased to a stable value with increasing running cycles. In the case of larger torsional angle, the torque also decreased during a few initial running cycles, and then it remained stable.

A plot of steady-state torsional friction torque against torsion angle is presented in Fig 2B. The torsion friction torque decreased with the torsion angle increasing until the angle reached 5°. When the torsion angle was larger than 5°, the torque maintained a correspondingly stable value. By performing a correlation analysis on the testing data, the following exponential relation between the torsion friction torque and the torsion angle was obtained:

$$T=0.059+0.045e^{-\theta /1.377},$$ (1)

where *T* represents the torsion friction torque (in units of Nm) and *θ* is the torsion angle (in units of degrees).

**Fig 3:**
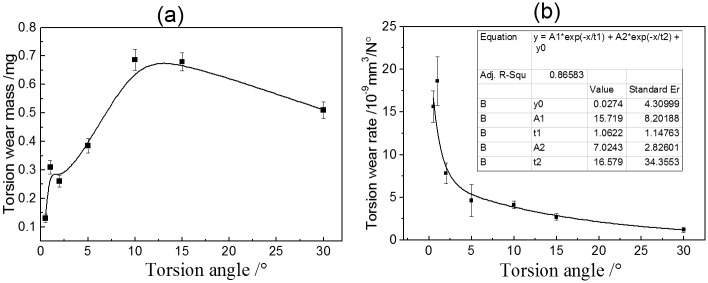
Variation of torsional wear mass loss and wear rate of PTFE as functions of torsion angle (a) Wear mass loss *v*s torsion angle; (b) Wear rate *v*s torsion angle.

Fig 3A and 3B show the wear of PTFE under different torsion angles. The wear mass loss of PTFE increased from 0.13 mg under a torsion angle of 0.5° to 0.69 mg under a torsion angle of 10°. After that, the wear mass loss decreased with increasing torsion angle. However, the wear rate of PTFE decreased when the torsion angle rose, as shown in Fig 3B. The following exponential relation between the wear rate of PTFE and the torsion angle was regressed:

$$W=0.027+15.720e^{-\theta /1.062}+7.024e^{-\theta /16.579},$$ (2)

where *W* represents the wear rate of PTFE (in units of 10^−9^ mm^3^/N°) and *θ* is the torsion angle.

### Effect of normal load

**Fig 4:**
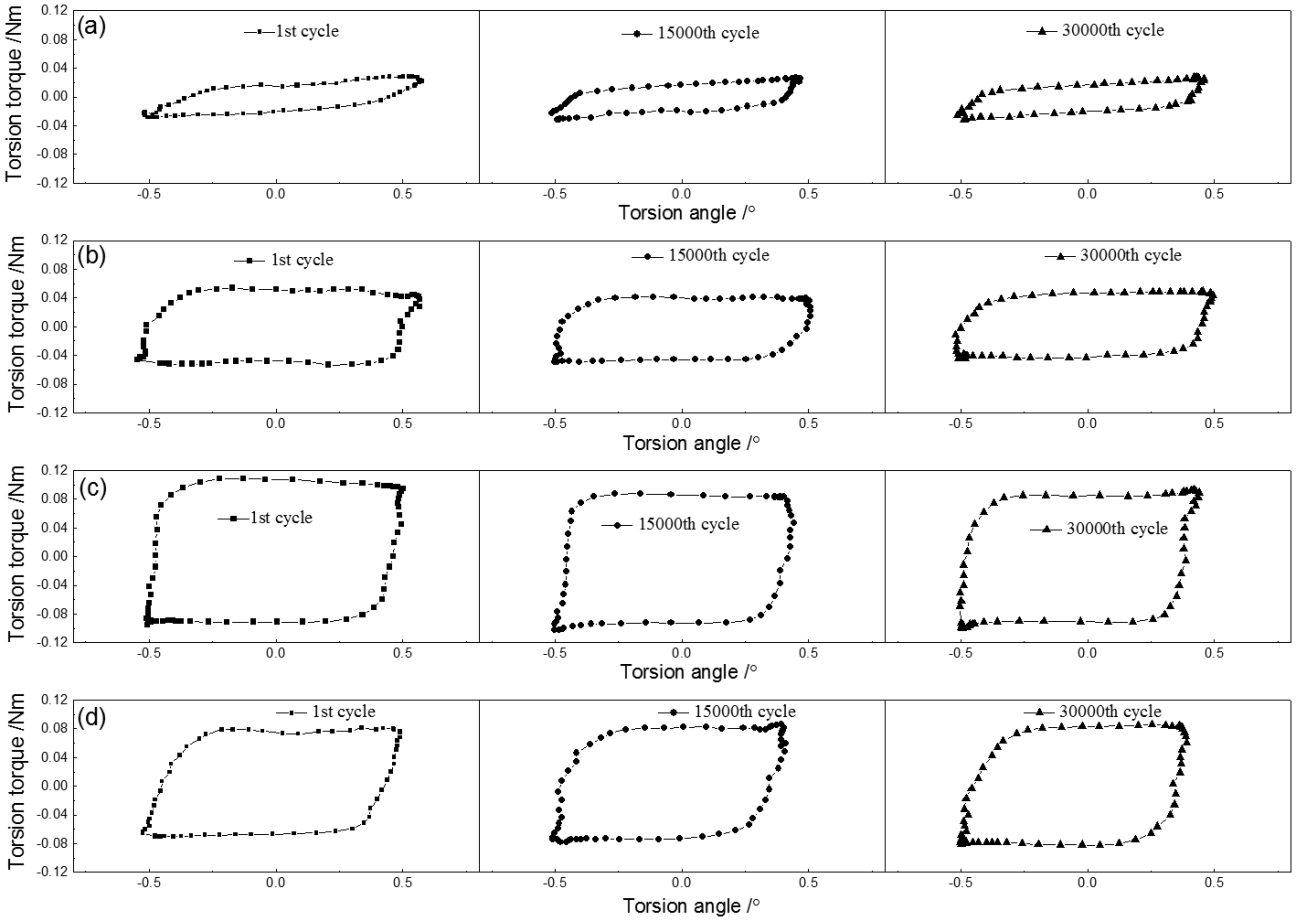
*T–θ* curves of PTFE under 1° torsion angle with different normal loads (a) *F* = 43 N,*θ =* 1°; (b) *F* = 83 N,*θ =* 1°; (c) *F* = 123 N,*θ =* 1°; (d) *F* = 163 N,*θ =* 1°.

**Fig 5:**
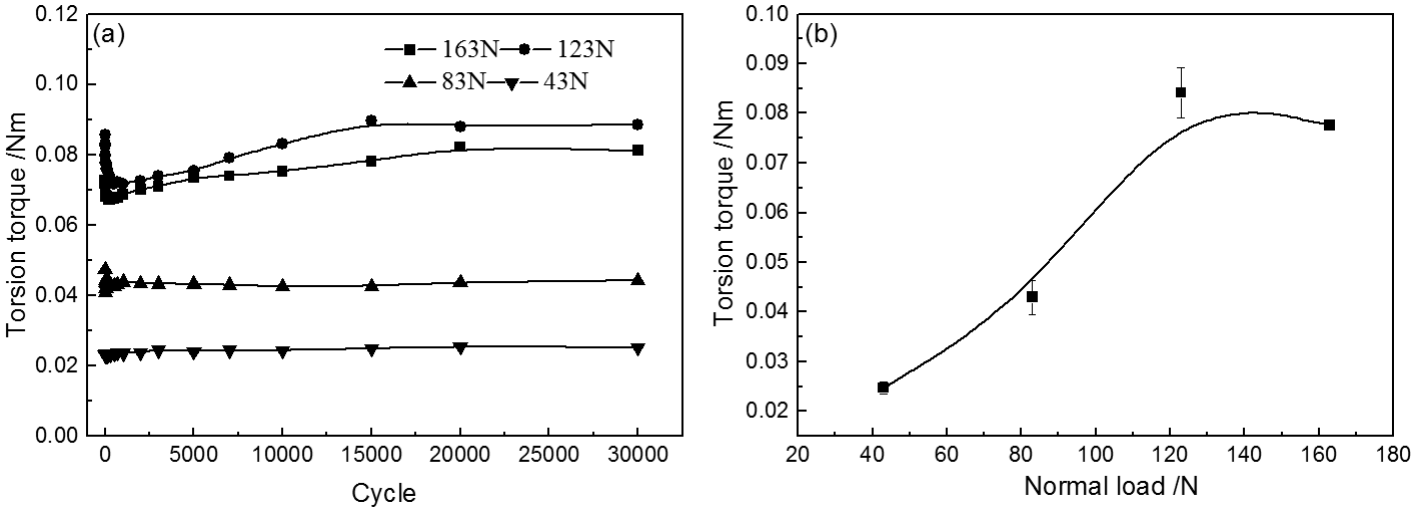
Torsional friction torque of PTFE under different normal loads. (A) Torsional friction torque versus different cycles and normal loads; (B) Torsional friction torque versus different normal loads.

**Fig 6:**
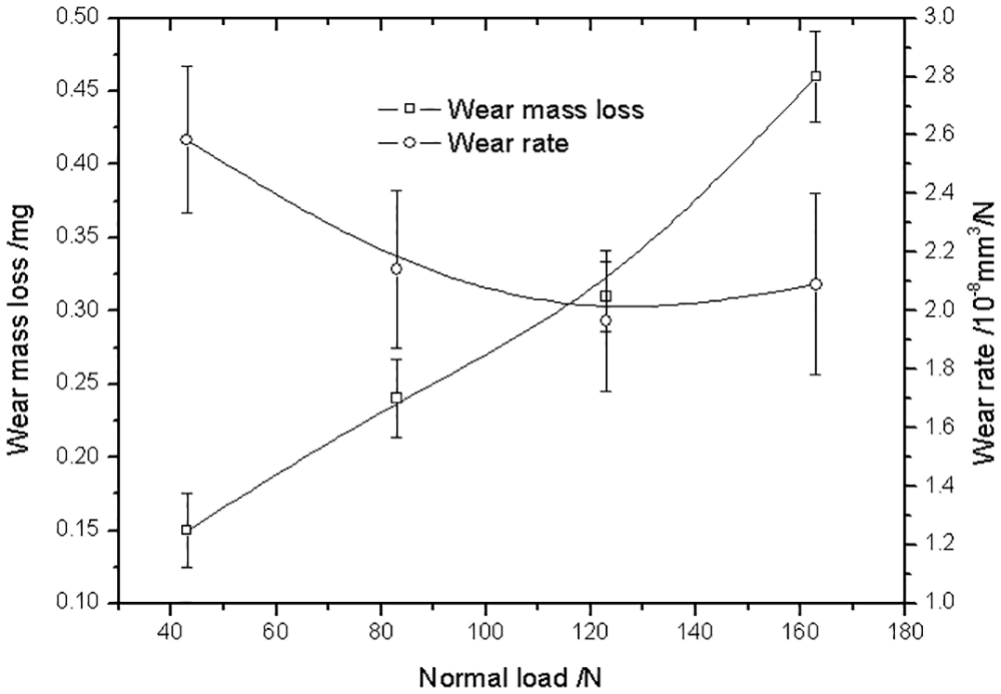
Variation of torsional wear of PTFE under different normal loads.

Fig 4A–4D present the *T–θ* curves of PTFE against a steel pin under 1° torsion angle displacement with different normal loads during torsional friction. The shape of *T–θ* curves was affected by the normal load. The *T–θ* curves exhibited a quasiparallelogrammatical shape under a 43 N normal load. Under other loads, the shape of *T–θ* curves was rectangular. In addition, the value of the torsion friction torque was also influenced by the normal load, as shown in Fig 5A and 5B. The torque increased from 0.025 N·m under a 43 N normal load to 0.082 N·m under a 123 N normal load. With sequentially increasing normal load, the value of the torque was maintained. With rising normal load, the wear mass loss of the PTFE disk was increased and the wear rate was decreased, as shown in Fig 6.

## Torsion Friction Model

**Fig 7:**
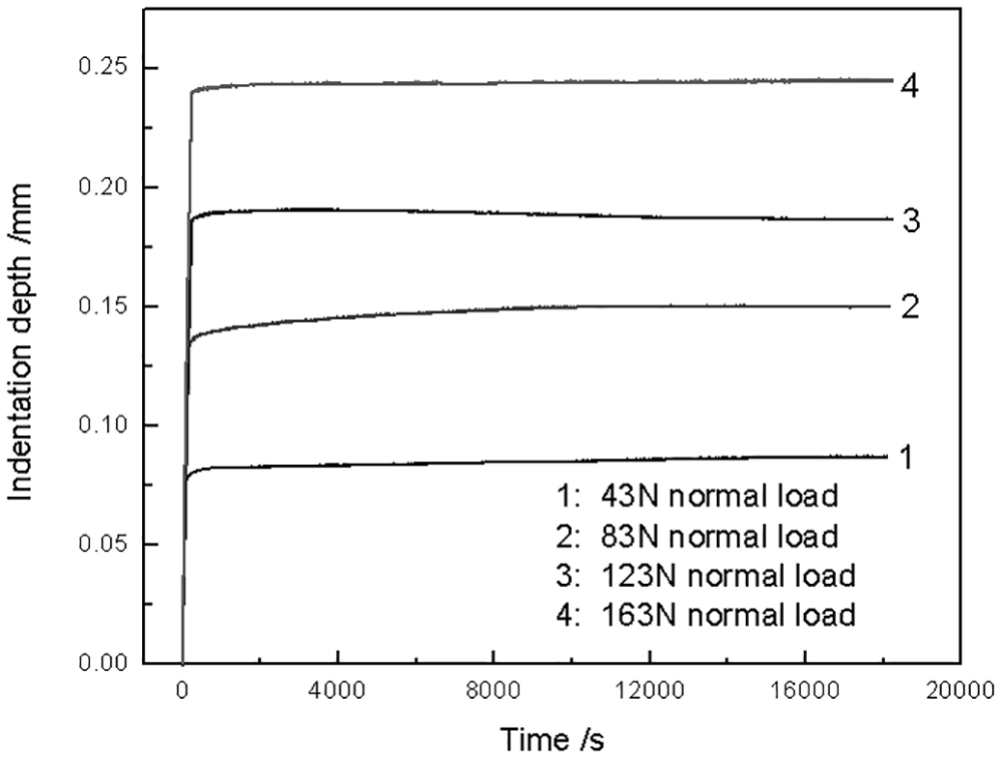
Variation of indentation depth of PTFE against time under different normal loads.

The torsional friction model was built based on contact mechanics. The nature of contact interaction included elastic, elastic-plastic, visco-elastic contact. So it was significant to ascertain a kind of contact nature in order to select a compatible contact model. PTFE is a kind of visco-elastic polymer and its stress/strain curve exhibits a linear increase below the yield stress (about 10MPa) [26]. The maximum among average pressure under the four testing loads was 2 MPa, which was lower than the yield strength of the PTFE. PTFE is a kind of visco-elastic material. The contact regime of PTFE was affected by its creep behavior. In order to research the effect of visco-elastic properties of PTFE on contact behavior, the indentation creep behavior of PTFE under four normal loads was measured, respectively. The curves of indentation depth are exhibited in Fig 7. It was noted that the indentation depth was maintained a constant value under the four normal loads. This indicated that the visco-elastic properties had little effect on the contact behavior under the lower loads. The elastic contact model was valid for the torsion contact.

With the development of computer technology, molecular dynamics (MD) and related atomistic methods for probing dynamical processes have been developed. Recently, an atomistic simulation method which allows the simulation at experimental time scale was developed by Timothy [27], and was applied to the creep study. In essence, creep is due to the results that the viscous resistance between macromolecules prevent the stress and deformation balance immediately. Analysis from the angle of molecular motion, the creep of polymer closely linked with the different forms of molecular motion [28, 29]. Under proper temperature and constant external loads, deformation of many small size units such as short chain-segment, lateral group, bond length and bond angle can instantly complete, and the deformation manifest as elastic deformation. Over time, chain-segments overcome the barrier of internal rotation and make freedom motion, the motion of chain-segments lead to the stretch of molecular chain, the deformation manifest as high elastic strain. The amount of high elastic strain is larger than elastic deformation, and the deformation can recovery when the external loads are removed. When the time is long enough, relative slippage occurs between fiber molecules and cause viscous flow, and the deformation is permanent deformation which cannot recovery after the external loads are removed.

In this paper, the flat indentation creep tests were conducted at room temperature (24°C) which is far lower than the glass-transition temperature (about 115°C). Under this temperature, the polymer is in glass state, the motion of chain-segment is difficult, but the activation energy which the motion of small size units such as short chain-segment, lateral group, bond length and bond angle needed is lower, therefore can be motivated by a small energy and make freedom movement [27]. Furthermore, under a lower stress condition (less than or equal to 2MPa), the relaxation time of chain-segment movement is very long, it is hard to observe the high elastic strain caused by the motion of chain-segment in a limited time. By this time, the creep deformation is mainly elastic deformation, the strain rate is very slow, and the amount of creep will not obvious over time. This analysis is also corresponds to the results showed in Fig 7. It was noted that the indentation depth was maintained a nearly constant value under the four normal loads. This means that creep is not obvious and the strain rates is close to zero. In other words, the visco-elastic property of PTFE has no effect on the experiment in this paper under the experiment condition of a temperature of 24°C and a lower stress of 2MPa.

A model of a rigid, flat-ended punch acting on an elastic half-space was adopted to described the torsional experiments because the elastic modulus of steel was much higher than that of PTFE. Johnson [18] and Jäger [22] systemlly analyzed the contact mechanics of a rigid, flat-ended punch acting on an elastic half-space. The following model was based on their results.

When a rigid, cylindrical flat-ended punch acts on an elastic half-space under a constant normal force, a uniform normal displacement occurs in the circle contact zone. The pressure distribution acting on an elastic half-space follows [18, 22]

$$p_{z}(r)=\{\begin{array}{c} 0\text{ for }r\geq a,\\ \frac{p_{0}}{\sqrt{a^{2}-r^{2}}}\text{ for }r\leq a,\; \end{array}$$ (3)

where *r* represents the radius, *a* is the radius of contact zone, and *P*_z_ is the pressure acting on a circle with a radius of *r*. The normal force *F*_z_ was obtained by integrating (3):

$$F_{z}=\int_{0}^{a}2\pi rp_{z}dr=2\pi ap_{0}.$$ (4)

When a small torsional torque acts on a rigid, flat-ended punch in contact with an elastic half-space, the solutions for the tangential stress *q*(*r*), the torsional angle *β*, the torsional moment *M*_z_, and the strain in polar coordinates (*μ*_θ_, *μ*_Z_, *μ*_r_), as outlined in [18, 22] under the condition of complete adhesion, are

$$q(r)=\frac{q_{0}r}{\sqrt{a^{2}-r^{2}}},\;\beta =\frac{\pi q_{0}}{4G},\;M_{z}=\frac{4}{3}\pi a^{3}q_{0}$$ (5)

for complete adhesion,

$$\mu_{\theta }=\left\{ \begin{array}{l} \frac{\pi q_{0}}{4G}r\text{ for }r\leq a,\\ \frac{q_{0}}{2G}\left(r{\text{sin}}^{-1}\frac{a}{r}-a\sqrt{1-\frac{a^{2}}{r^{2}}}\right)\text{ for }r\geq a, \end{array}\right.$$ (6)

$$\mu_{z}=\mu_{r}=0.$$ (7)

Since the tangential stress is infinite for *r* = *a*, slip must take place, starting at the border of the contact area. The basic idea of the general solution presented by Jäger [22] is to superpose differential rigid punch solutions, which are functions of the punch radius *a*, to arrive at the solution for partial sliding. The solution must satisfy Coulomb’s law in the sliding area, and it must describe rigid rotation in the area of adhesion. The circumferential stress in the sliding area and in the adhesion area can be calculated from (5) as

$$\sigma_{z\varphi }=\left\{ \begin{array}{l} \int_{r}^{a}\frac{rq_{0}(s)}{\sqrt{s^{2}-r^{2}}}ds=fP_{\text{z}}\text{ for }a\geq r\geq a*\\ \int_{a*}^{a}\frac{rq_{0}(s)}{\sqrt{s^{2}-r^{2}}}ds\text{ for }r\leq a* \end{array}\right.,$$ (8)

where a* is the adhesion radius that divides the sliding area and adhesion area, *f* is the coefficient of friction, and *σ*_*zφ*_ is the circumferential stress.

The torsional angle is the same constant in both sliding and adhesion areas. Thus the torsional angle *β* can be obtained as a superposition of the function in the sliding area:

$$\beta (a*,a)=\frac{\pi }{4G}\int_{a*}^{a}q_{0}(s)ds.$$ (9)

For prescribed normal pressure *p*_*z*_, the Abel integral eq (8) can be inverted, with the result

$$q_{0}(s)=-\frac{2f}{\pi }\frac{d}{ds}\int_{s}^{a}\frac{p_{z}(a,x)}{\sqrt{x^{2}-s^{2}}}dx.$$ (10)

Insertion of Eq (10) in Eq (9) gives the following result for *β*:

$$\beta (a*,a)=\frac{f}{2G}\int_{a*}^{a}\frac{p_{z}(a,x)}{\sqrt{x^{2}-a{*}^{2}}}dx$$ (11)

The functional relationship between the torsional angle *β* and the radius of the sliding area is obtained through Eq (11) for a torsional friction pair with a uniform coefficient of friction under a defined normal load.

Insertion of Eq (10) in Eq (5) gives the torsional torque in the adhesion area:

$$M_{z}(0,a*)=\frac{4\pi }{3}\int_{0}^{a*}s^{3}(-\frac{2f}{\pi }\frac{d}{ds}\int_{s}^{a}\frac{p_{z}(a,x)}{\sqrt{x^{2}-s^{2}}}dx)ds.$$ (12)

The torsional torque in the sliding area can be calculated according to Coulomb’s law as

$$M_{z}(a*,a)=\int_{a*}^{a}2\pi fp_{z}rds=f\int_{a*}^{a}\frac{F_{z}}{a\sqrt{a^{2}-r^{2}}}r^{2}dr.$$ (13)

Therefore the total torsional torque *M*_*z*_ is

$$M_{z}=M_{z}(0,a*)+M_{z}(a*,a).$$ (14)

## Discussion

**Fig 8:**
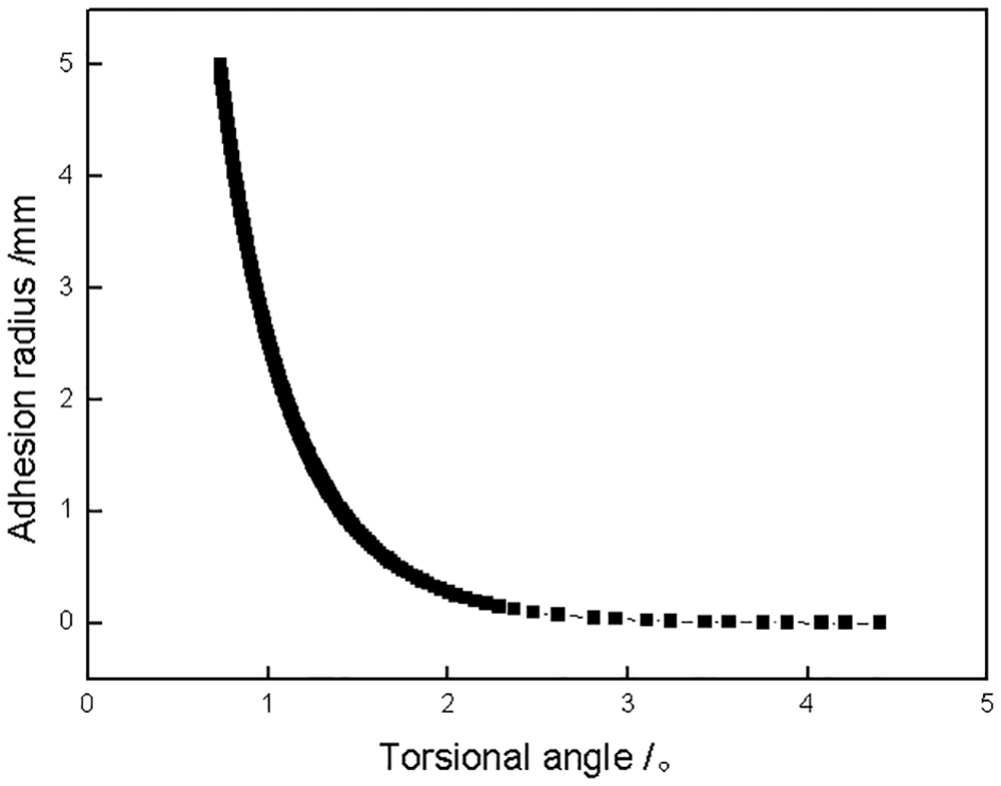
Adhesion radius of the torsional contact pair as a function of torsional angle under a 123 N normal load.

In this section, the results are calculated according to the experimental conditions. The coefficient of sliding friction for PTFE against 1045 steel is 0.12, as determined from a sliding friction test [30]. For the quantitative analysis of the transition of the contact regime (adhesion or slip), Eq (11) could easily be calculated to obtain the torsional angles as a function of adhesion radius. Then the calculated result was arbitrarily inverted and regenerated in Fig 8. Fig 8 shows the variation of adhesion radius of the torsional contact pair as a function of different torsional angles under a 123 N normal load. The adhesion radius of the torsional contact pair first decreased rapidly to about zero when the torsional angle increased to 2°, indicating that the adhesion area decreased and the slip area increased with increasing torsional angle. When the torsional angle was lower than 2°, the partial slip regime was the main torsion kinematics behavior in the contact zone. Gross slip occurred in the torsional contact zone when the torsional angle was larger than 2°.

**Fig 9:**
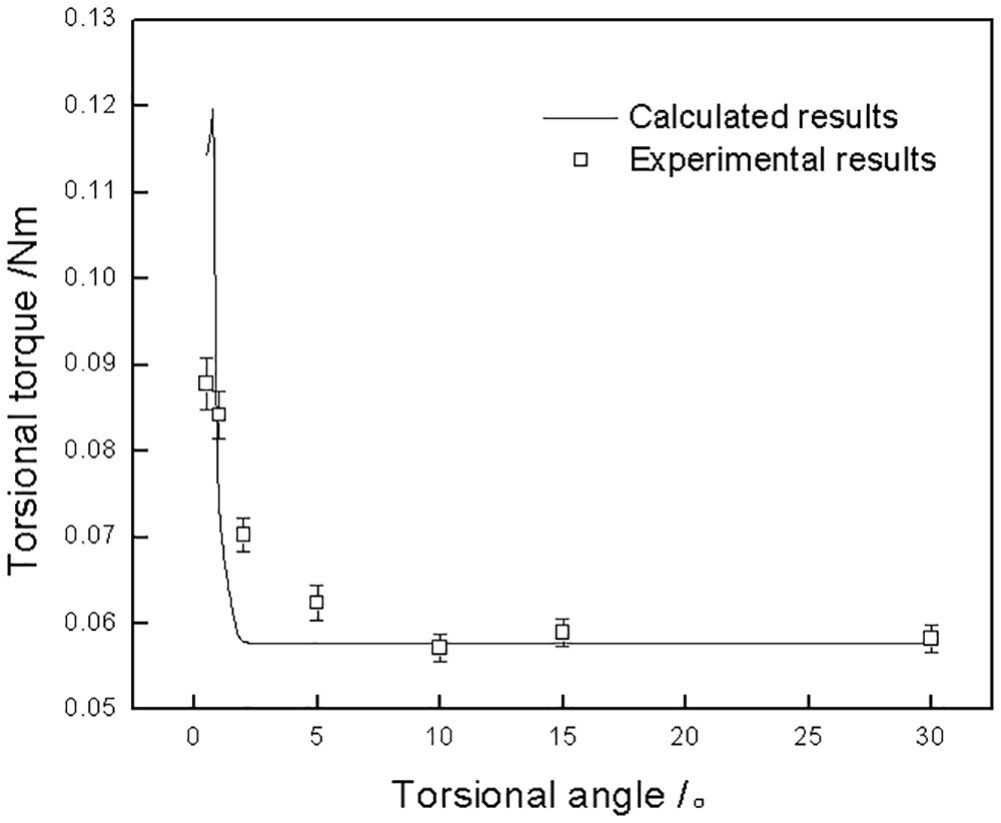
Torsional torque of torsional contact pair as a function of torsional angle under a 123 N normal load.

The variation of calculated torsional torque against torsional angle is presented in Fig 9. Similar to adhesion radius, the torsional torque also decreased sharply with rising torsional angle until the angle increased to 2°. After that, the torsional torque maintained a correspondingly stable value (0.058 N·m). When the torsional angle was larger than 2°, gross slip occurred, and the torque was scarcely affected by the torsional angle. This is similar to reciprocating friction, in which the reciprocating distance has little effect on the tangential friction force. Compared to the variation of adhesion radius shown in Fig 8, it can be deduced that the torsional kinematics (adhesion, partial slip, and gross slip) was the main factor influencing the torsional torque. The torque of torsional contact under partial slip was higher that that under gross slip, and the torque under partial slip decreased with decreasing adhesion area (adhesion radius). The experimental torque is also plotted in Fig 9. The calculated results are in good agreement with the experimental results. This indicates that the torsional friction model is valid for predicating the torsional torque of PTFE.

**Fig 10:**
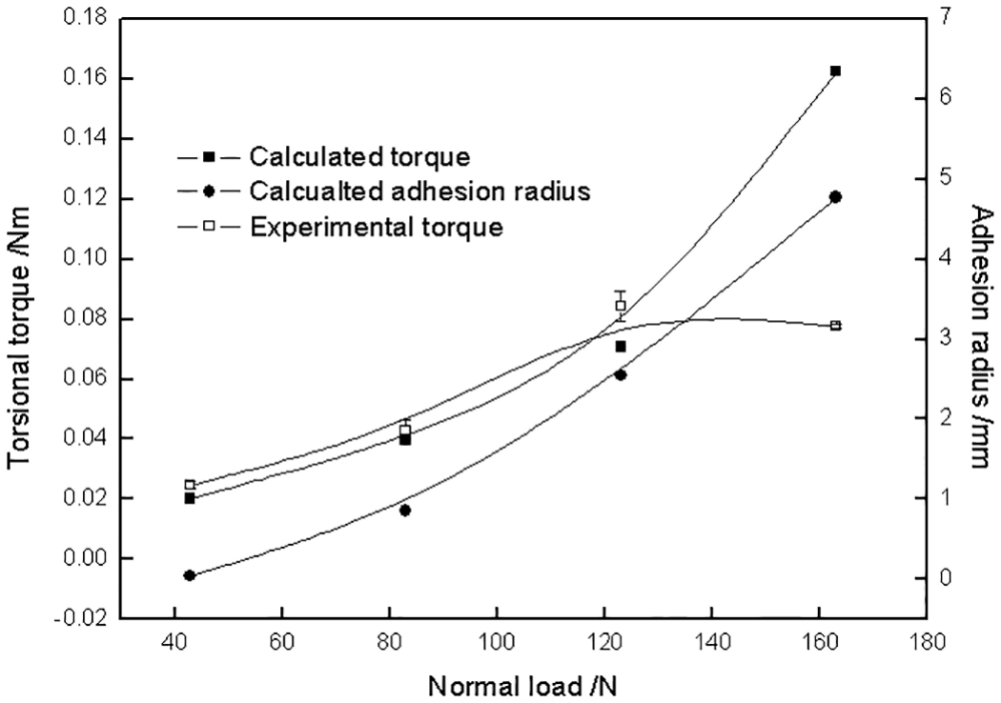
Variation of torsional torque and adhesion radius of torsional contact versus normal load under a 5° torsional angle.

Fig 10 shows the variation of calculated torsional torque against different normal loads under 1° torsional angle. Both the calculated torque and adhesion radius increased with increasing normal load. The experimental torque is seen to be close to the calculated value except for that under a 163 N normal load. The difference between experimental and calculated torque under a 163 N normal load was caused by the coefficient of friction. Usually the coefficient of friction of a polymer decreases with increasing normal load, whereas a constant coefficient of friction was applied in the model.

## Conclusions

In this work, the plane-on-plane torsional tribological behavior of PTFE was studied. The shape of the *T–θ* curves was influenced by the torsional angle: rectangular under the larger torsional angle displacements (30° and 15°) and quasiparallelogrammatical under the smaller torsional angle displacements (1° and 0.5°), respectively. The torsion friction torque decreased with increasing torsion angle until the angle reached 5°. When the torsion angle was larger than 5°, the torque maintained a correspondingly stable value. An exponential relation between the torsion friction torque and the torsion angle was regressed. The wear rate of PTFE exponentially decreased when the torsion angle rose. The torque increased from 0.025 N·m under a 43 N normal load to 0.082 N·m under a 123 N normal load. By increasing normal load sequentially, the value of torque was maintained. With rising normal load, the wear mass loss of the PTFE disk was increased and the wear rate was decreased.

A model of a rigid flat-ended punch acting on an elastic half-space was built. Based on the model, the adhesion radius and the torsional torque both decreased with increasing torsional angle. The plot of calculated torque versus torsional angle agreed well with the experimental results. When the normal load increased, the calculated torque also increased. The experimental torque was close to the calculated value except for a difference under 163 N normal load, attributed to a uniform coefficient of friction being used in the model.
